# A Universal Method for Species Identification of Mammals Utilizing Next Generation Sequencing for the Analysis of DNA Mixtures

**DOI:** 10.1371/journal.pone.0083761

**Published:** 2013-12-16

**Authors:** Andreas O. Tillmar, Barbara Dell'Amico, Jenny Welander, Gunilla Holmlund

**Affiliations:** 1 Department of Forensic Genetics and Forensic Toxicology, National Board of Forensic Medicine, Linköping, Sweden; 2 Department of Clinical and Experimental Medicine, Faculty of Health Sciences, Linköping University, Linköping, Sweden; Erasmus University Medical Center, Netherlands

## Abstract

Species identification can be interesting in a wide range of areas, for example, in forensic applications, food monitoring and in archeology. The vast majority of existing DNA typing methods developed for species determination, mainly focuses on a single species source. There are, however, many instances where all species from mixed sources need to be determined, even when the species in minority constitutes less than 1 % of the sample. The introduction of next generation sequencing opens new possibilities for such challenging samples. In this study we present a universal deep sequencing method using 454 GS Junior sequencing of a target on the mitochondrial gene 16S rRNA. The method was designed through phylogenetic analyses of DNA reference sequences from more than 300 mammal species. Experiments were performed on artificial species-species mixture samples in order to verify the method’s robustness and its ability to detect all species within a mixture. The method was also tested on samples from authentic forensic casework. The results showed to be promising, discriminating over 99.9 % of mammal species and the ability to detect multiple donors within a mixture and also to detect minor components as low as 1 % of a mixed sample.

## Introduction

Determination of unknown species is of interest in many areas of applied genetics. For example, in forensic genetics, determination of species can be used to aid police investigations for the identification of forensic stains [1], solve cases of poaching [2,3] and aid the protection against trading with endangered species [4,5]. In food industry the authenticity of meat or fish species can be monitored [6-8] and in archeology human remains can be sorted from non-human remains [9]. 

The majority of existing DNA typing methods for species determination focuses on single-species DNA sources and is based on PCR amplification using species-specific primers. There are, however, many instances were no a priori information about the species is available. For such cases a universal typing method would be valuable, especially if the method has the possibility to detect several species in a mixed source. Example of such issues could be bite marks [10], meat or fish in food [11] or in fact any contaminated samples where the source in minority is of interest. 

During the recent years there has been an enormous progress in DNA sequencing technologies. These so called next generation sequencing (NGS) technologies have by massively parallel and clonal sequencing increased the ability to gain sequence information from single molecules within a complex or degraded DNA source. These new technologies have, for example, been used for large genome sequencing projects such as the typing of the Neanderthal and the woolly mammoth genomes [12,13]. NGS technologies have also been used in more specified projects either using a metagenomic shot gun sequencing approach [1] or a more targeted approach [14,15]. In applications for species identification, Coghlan and colleagues [16] recently presented a cost-effective and efficient method using a next generation deep sequencing technology for the identification of plants and animals in traditional Chinese medicines.

The above mentioned studies have been performed at research laboratories with access to research-intensive high-throughput sequencing technologies [17]. The recent and fast development of bench top next generation sequencers like the 454 GS Junior (Roche), Ion Torrent (Promega) and MiSeq (Illumina) have made NGS technologies applicable and affordable for people working in different fields of applied genetics [18]. 

Encouraged by these new technologies, we present a DNA-typing method for the determination of mammal species using targeted massively parallel sequencing of a short mitochondrial DNA (mtDNA) sequence utilizing the benefits of the NGS bench top technologies. Some of the major advantages with the method are: 1) Short PCR amplicon, i.e. facilitating the analysis of degraded and poor DNA sample quality; 2) Universal PCR-primers, i.e. there is no need for a priori species information; 3) Economy, i.e. the high capacity for sample multiplexing reduces cost per sample; and 4) Deep sequencing, i.e. the possibility to detect DNA present in minute amounts. 

The outline of the paper is as follows. The design of the target sequence is presented including a phylogenetic analysis of mitochondrial DNA sequences from more than 300 species belonging to the Class Mammalia After this in silico design, the selected universal PCR primers were tested in vitro for their ability to generate PCR-amplicons with DNA from several different species. Experiments were furthermore performed on artificially mixed DNA samples using the NGS technology 454 GS Junior (Roche) to detect all species within an artificial mixture. Finally, the method was tested on two samples from authentic forensic casework.

## Materials and Methods

### Design of the target and phylogenetic analyses

The complete genome of the mitochondria for 334 different species (Table S1) belonging to the Class Mammalia were downloaded from GenBank (http://www.ncbi.nlm.nih.gov/nuccore) and the DNA sequences for the 16S ribosomal RNA gene were extracted using the FeatureExtract 1.2 Server (http://www.cbs.dtu.dk/services/FeatureExtract). Multiple alignment was performed by the means of MAFFT (http://mafft.cbrc.jp) using the default settings. From the resulting alignment, the sequences for the universal PCR primers, designed previously [19], were extracted together with the DNA sequence between the forward and reverse primer sites (i.e. the target region). A distance matrix was computed by pairwise comparison of the DNA sequences between the primer sites for all 334 mammal species, using MEGA v. 5.1 [20]. In order to visualize the ability to separate different mammal species, a tree (UPGMA) was created using MEGA v. 5.1. The tree was constructed based on the total number of nucleotide differences for the complete target region between the 334 mammal reference sequences. Note that only one sequence was included for each species and thus intraspecific variability was not considered (see the discussion for further comments on this).

### Verification tests

A number of different analyses were performed on DNA samples of known origin and DNA samples from authentic forensic casework to test the method and its ability to be applied in routine:

**Table 1:** DNA-sequence for 15 mammal species using the universal primers for DNA amplification.

**Species**	**DNA sequence of the target region (5'-3')**
Harbour seal (*Phoca vitulina*)	TTTAATTAACTAACTCAACAGAGCAAATCCAGTCAACCAACAGGGAATAAAAACTTCTACAATGAGTTAGCAATTTA
European beaver (*Castor fiber*)	TTTAATTTTTCAACCACAATTATACAATAACTCCTACCCCTAATTGGCCCAACCCTATAATTTCtGGTTTAATAATTTT
Brown bear (*Ursus arctos*)	TTCAATTAATTAGCTCAAAAGGATTTATTTACCAGACCGACAGGAACAACATATTCCTTCCATGAGCTAGCAATTTA
Red fox (*Vulpes vulpes*)	TTTAATTAATTAGCCCAAACTTATGAACTTTAAACCCCACTGGGAATAACATACTACCATTGTTATGGGCTGACAATTTA
Otter (*Lutra lutra*)	TTTAATTAACTAACCCATAATAACTTACTAAATCACCGATCAGGCCTAACACAATCCTATTAATGGGTTAGCAATTTA
Lynx (*Lynx lynx*)	TTTAATTAACCGACCCAAAGAGACCCCATTTATCCAACCGACAGGAACAACAAACCTCCACTATGGGTCGACAATTTA
Wolverine (*Gulo gulo*)	TTTAATTAACTAACCCATAATAAGTCTACTTAATCACCAACCGGGTCTAACACAACCTTATTAATGGATTAGTAATTTA
Pig (*Sus scrofa*)	TTTAATTAACTATTCCAAAAGTTAAACAACTCAACCACAAAGGGATAAAACATAACTTAACATGGACTAGCAATTTC
Horse (*Equus caballus*)	TTTAATTAACTGATTCACAAAAAACAATACACAAACCTAACCTTCAGGGACAACAAAACTTTTGATTGAATCAGCAATTTC
Cow (*Bos taurus*)	TTTAACTAACCAACCCAAAGAGAATAGATTTAACCATTAAGGAATAACAACAATCTCCATGAGTTGGTAGTTTC
Ringed seal (*Phoca hispida*)	TTTAATTAACTAACTCAACAGAACAAATCCAGTCAACCAACAGGGAATAAAAACTTCTATAATGAGTTAGCAATTTA
Porpoise (*Phocoena phocoena*)	TTTAATTAATCAACTCAAAAAAATCGTAAAACAGTACCACTAAGGGATAACAAAATTTTATATGGGTTGACAATTTC
Polecat (*Mustela putorius*)	TTCAATTAACTAACCCACAATAACCAATCAATATGCCAACCAGGCCTAACATAATCTTATTTCTGGGTTAgcAATTTA
Wolf (*Canis lupus*)	TTTAATTAACTAACCCAAACTTATGGATACTAGATACCTACAAGGCATAACATAACACCATTATTATGGGTTAGCAATTTA
Human (*Homo sapiens*)	TTTAATTTATTAATGCAAACAGTACCTAACAAACCCACAGGTCCTAAACTACCAAACCTGCATTAAAAATTTC

**Table 2:** Summary of the analyses of artificial DNA mixtures.

**Mixture ratio**	**Species 1: Species 2**	**All species detected?**	**Total number of reads after filtering**
1:1	Dog (*Canis lupus familiaris*): Human (*Homo sapiens*)	Yes	1,645
1:1	Elk (*Alces alces*): Human (*Homo sapiens*)	Yes	2,958
1:1	Deer (*Dama dama*): Elk (*Alces alces*)	Yes	2,105
1:1	Bear (*Ursus arctos*): Human (*Homo sapiens*)	Yes	772
1:1	Wild boar (*Sus scrofa*): Human (*Homo sapiens*)	Yes	1,559
1:1	Elk (*Alces alces*): Human (*Homo sapiens*)	Yes	23,055
1:1	Elk (*Alces alces*): Human (*Homo sapiens*)	Yes	11,527
1:1:1:1	Roe deer (*Capreolus capreolus*): Elk (*Alces alces*): Bear (*Ursus arctos*): Dog (*Canis lupus familiaris*)	Yes	6,594
99:1	Elk (*Alces alces*): Dog (*Canis lupus familiaris*)	Yes	16,354
99:1	Dog (*Canis lupus familiaris*): Bear (*Ursus arctos*)	Yes	22,473
99:1	Bear (*Ursus arctos*): Elk (*Alces alces*)	Yes	32,975
99:1	Cow (*Bos taurus*): Pig (*Sus scrofa domesticus*)	Yes	92,352
99:1	Elk (*Alces alces*): Human (*Homo sapiens*)	Yes	18,689
99:1	Deer (*Dama dama*): Elk (*Alces alces*)	Yes	1,665
99:1	Dog (*Canis lupus familiaris*): Human (*Homo sapiens*)	Yes	2,405
99:1	Human (*Homo sapiens*): Wild boar (*Sus scrofa*)	Yes	7,420
99:1	Human (*Homo sapiens*): Wild boar (*Sus scrofa*)	Yes	15,711
99:1	Bear (*Ursus arctos*): Human (*Homo sapiens*)	No, only Bear	1,474
99:1	Elk (*Alces alces*): Human (*Homo sapiens*)	No, only Elk	3,069
99:1	Elk (*Alces alces*): Human (*Homo sapiens*)	No, only Elk	11,165
99:1	Wild boar (*Sus scrofa*): Human (*Homo sapiens*)	No, only Wild boar	1,392

1) In order to verify the specificity of the universal PCR primers, DNA from 15 different species of known origin (Table 1) was amplified and sequenced, individually with Sanger sequencing as described below. The samples were provided by The Swedish National Veterinary Institute and The Swedish Museum of Natural History [19]. 2) Twenty two-species DNA mixtures and one four-species DNA mixture (Table 2 and Table S2), with known DNA template copy number ratios, were analyzed with deep sequencing using GS 454 Junior (Roche) as described in detail below. 3) The 454 deep sequencing method was applied on five samples from authentic forensic casework. The task was to detect the minor component of assumed DNA mixtures in all samples. The first sample was taken from a case with a man accused of animal cruelty of a dog. The question was if DNA from dog could be detected in the trace sampled from the man. The remaining samples were from a case with a human corpse that had multiple bite marks of an unknown “attacker”; here the question was “which species made the bite marks?” 

### DNA extraction, mtDNA copy quantification and artificial mixtures

For the reference samples, DNA were extracted based on a previously described method [21], and the DNA from the two authentic case samples was extracted using a Chelex method [22]. The number of mtDNA copies in a DNA extraction was quantified using an in-house developed assay based on quantitative real time PCR. The quantification method utilized SYBR Green I chemistry using the same primer pair as for the 454 PCR amplification protocol described below (Table 3). Each PCR reaction contained 12.5 μl 2x QuantiFast SYBR Green PCR Master Mix (Qiagen, UK), 1 µl of each primer (800 nM final conc.), 8.5 μl RNase-free water and 2 μl of DNA template for a total reaction volume of 25 μl. Thermal cycling was performed in an iQ™5 multicolor Real-Time PCR Detection System (Bio-Rad) using conditions recommended in the QuantiFast protocol (Qiagen). Plasmids (GenExpress, Germany), with a known number of copies, containing the targeted DNA sequence for humans, were used as a standard reference. All samples were quantified in duplicate and the mean value was used. 

**Table 3:** PCR primer sequences.

**Application**	**Primer**	**Sequence (5’ to 3’**)
454-sequencing	Forward PCR primer	cgtatcgcctccctcgcgccatcagxxxxxxxxxxgacgagaagaccctatggagcGACGAGAAGACCCTATGGAGC
454-sequencing	Reverse PCR primer	ctatgcgccttgccagcccgctcagxxxxxxxxxxtccgaggtcrccccaaccTCCGAGGTCRCCCCAACC
Sanger sequencing	Forward PCR primer	tgtaaaacgacggccagtGACGAGAAGACCCTATGGAGC
Sanger sequencing	Reverse PCR primer	caggaaacagctatgaccTCCGAGGTCRCCCCAACC

xxxxxxxxxx is the barcode, and the nucleotides in upper case are the primer sequence that anneals to the target gene.

Pooled DNA samples from different species were used to create artificial DNA mixtures either in a 1:1 or in a 99:1 mtDNA copy number ratio.

### Sanger sequencing and 454 sequencing

For the Sanger sequencing, the targeted DNA sequence was amplified using the PCR primers, presented in Table 3, tailed with M13-adaptors. The PCR amplicons were sequenced by standard Sanger sequencing using BigDye® Direct Cycle Sequencing kit (Applied Biosystems) according to the manufacturer’s protocol. Separation, by capillary electrophoresis, was performed on an ABI3500 XL instrument using POP-7 (Applied Biosystems) and the DNA sequences were called using Sequence analysis software v. 5.4 (Applied Biosystems).

For the 454 deep sequencing, the HPLC-purified PCR primers (Biomers) were used with adaptors suitable for the 454 sequencing chemistry on the 454 GS Junior (Roche) (Table 3). Barcodes were included in the primers to aid the ability for sample multiplexing (Table S3). The DNA was amplified using an in-house developed assay as follows. Each PCR reaction contained GeneAmp 10x PCR Buffer (Applied Biosystems), 3 mM MgCl_2_, 0.5 mM Nucleotide+Uracil, 2 % Glycerol, 0.016 % BSA, 1.25 U AmpliTaq Gold DNA polymerase (Applied Biosystems), 0.05 U Uracil-DNA Glycosylase (USB corporation), 800 nM of each primer and 1 μl of DNA template (1,000-10,000 mtDNA molecules), for a total reaction volume of 25 μl. Thermal cycling was performed in a GeneAmp^®^ PCR System 9700 (Applied Biosystems) with touch-down PCR using the following parameters: 10 min at 37 °C, 5 min at 95 °C, followed by 10 cycles of 30 s at 94 °C, 30 s at 67 °C (minus 1 °C per cycle), 30 s at 72 °C and 30 cycles of 30 s at 94 °C, 30 s at 58 °C, 30 s at 72 °C with the final extension for 10 min at 72 °C. 

The PCR products were purified using AMPure (Beckman Coulter) following the manufacturer’s protocol. The PCR products were then quantified using KAPA SYPR^®^ FAST Bio-Rad iCycler qPCR kit (KAPABiosystems) according to the manufacturer’s protocol. Thermal cycling was performed in an iQ™5 multicolor Real-Time PCR Detection System (Bio-Rad) using conditions recommended by the manufacturer. All samples were quantified in duplicates and the final PCR product was diluted to 10^8^ molecules per µl. 

For the emulsion PCR (emPCR) and sequencing on 454 GS Junior, the manufacturer’s protocols were followed with reduced volume of primers (1/2 of the suggested volume) and the addition of 20 molecules per bead compensating for the short length of the PCR amplicon. 

### Bioinformatics

Sff-files extracted from the 454 GS Junior instrument were converted to FASTQ-files using the web-based tool Galaxy (https://main.g2.bx.psu.edu/). Reads with low quality were removed followed by the separation of the reads originating from different PCR:s by filtering on the barcode sequences. The barcodes were further removed, together with primer sequences, from the reads using Tagcleaner (http://edwards.sdsu.edu/cgi-bin/tagcleaner/tc.cgi). The resulting reads were grouped by collapsing identical reads. Sequences with less then 20 identical reads were removed from further analysis unless otherwise stated. The remaining sequences were searched against sequences in GenBank using BLAST (http://blast.ncbi.nlm.nih.gov/Blast.cgi), in order to identify the origin of the unknown DNA-sequences. Data described herein is available from the Sequence Read Archive (SRA) with the accession number SRA107543.

## Results

### Design of the target and phylogenetic analyses

The DNA sequences for all 334 mammal species were aligned and the primer site sequences were extracted and compared with the universal primer sequences (Table 3, modified from [19]). There were high homologies among the reference species for the majority of the nucleotide positions, both for the forward and the reverse primer sequences (Table 4). Further analysis showed that 97 % of the sequences from the reference species had primer sequences that were identical, or only with one nucleotide difference, to the universal primer.

**Table 4:** The compositions (in %) of nucleotides among the 334 reference sequences for the forward (A) and the reverse (B) universal primers.

A																						
Nucleotide																						
A	0	100	0	0	100	0.3	100	100	0	100	0	0	0	0	87.4	0	0	0.3	100	0.3	0	
G	100	0	0	100	0	99.7	0	0	100	0	0	0	0	0	12.3	0	100	99.7	0	99.7	0	
C	0	0	100	0	0	0	0	0	0	0	100	100	99.7	0.6	0	2.1	0	0	0	0	100	
T	0	0	0	0	0	0	0	0	0	0	0	0	0	99.4	0.3	97.9	0	0	0	0	0	
-	0	0	0	0	0	0	0	0	0	0	0	0	0.3	0	0	0	0	0	0	0	0	
Forward primer (5’.3’)	G	A	C	G	A	G	A	A	G	A	C	C	C	T	A	T	G	G	A	G	C	
B																						
Nucleotide																						
A	0	0	0	0	99.1	0.3	0.3	0	0	85.9	0	0	0	0	100	100	0	0	0	0	0	0
G	0	0	0	100	0	99.7	99.7	0	0	13.8	0	0	0	0	0	0	0	0	0	0	0	100
C	1.2	100	99.7	0	0.9	0	0	0.3	100	0.3	100	100	100	100	0	0	99.7	99.4	1.2	100	99.7	0
T	98.8	0	0.3	0	0	0	0	99.7	0	0	0	0	0	0	0	0	0.3	0.6	98.8	0	0.3	0
-	0	0	0	0	0	0	0	0	0	0	0	0	0	0	0	0	0	0	0	0	0	0
Reverse primer (5’.3’)	T	C	C	G	A	G	G	T	C	R	C	C	C	C	A	A	C	C	T	C	C	G

The nucleotides in majority were as follows for the forward primer; gacgagaagaccctatggagC (5’-3’) and tccgaggtcAccccaaccTCCG (5’-3’) for the reverse primer, which are identical to the primer sequences used for the PCR amplification (Table 3). Thus, based on this phylogenetic study, the selected universal primer sequences should be able to produce PCR amplicons for further analysis for the largest proportion of mammal species. 

**Figure 1:**
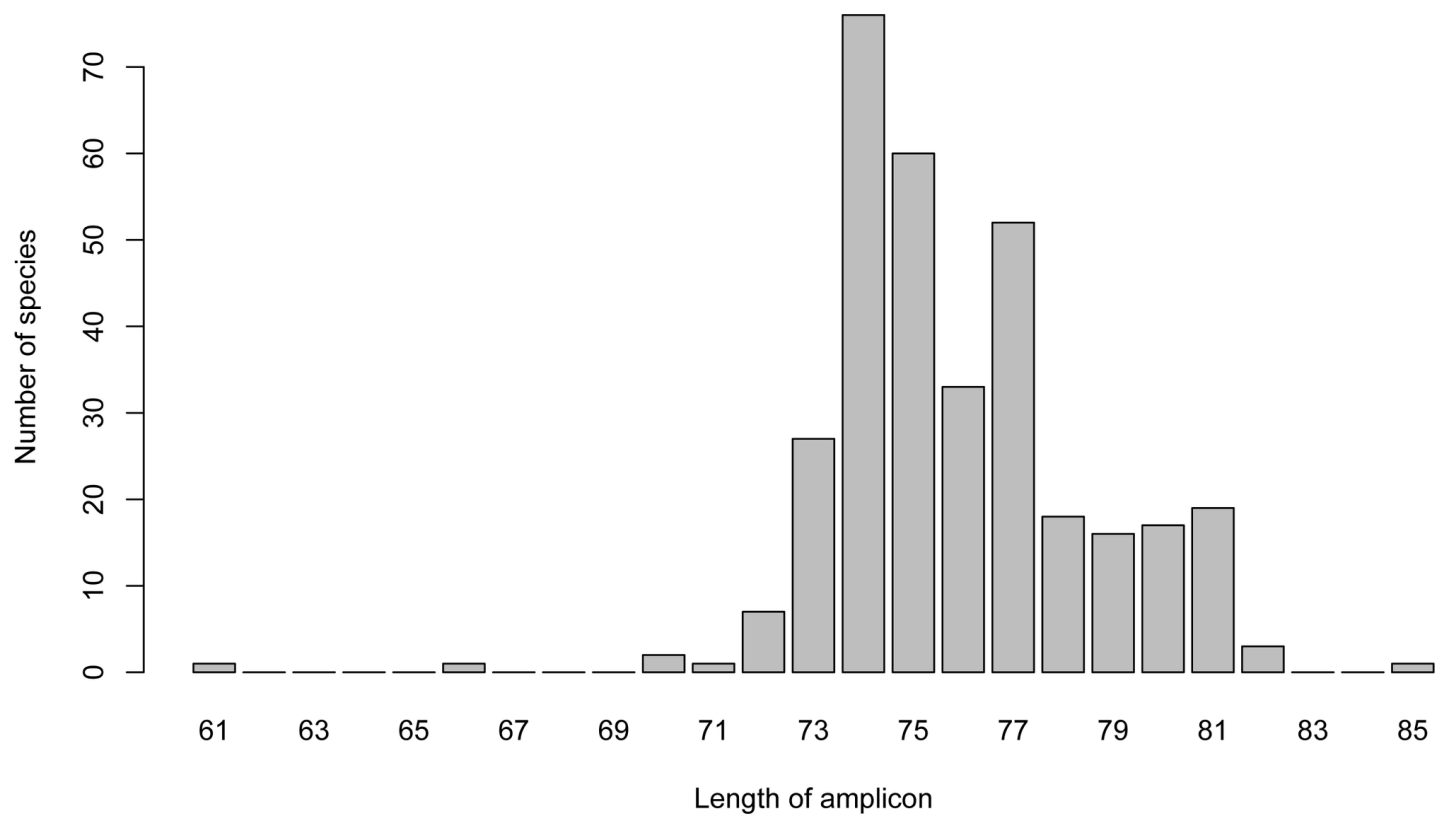
Distribution of the length (in base pairs) of the target sequence among 334 mammal species.

Further analysis showed that the length of the DNA sequence between the primer sites varied (Figure 1) among the reference species, with an overall mean of 75 base pairs (SD = 3 bp).

**Figure 2:**
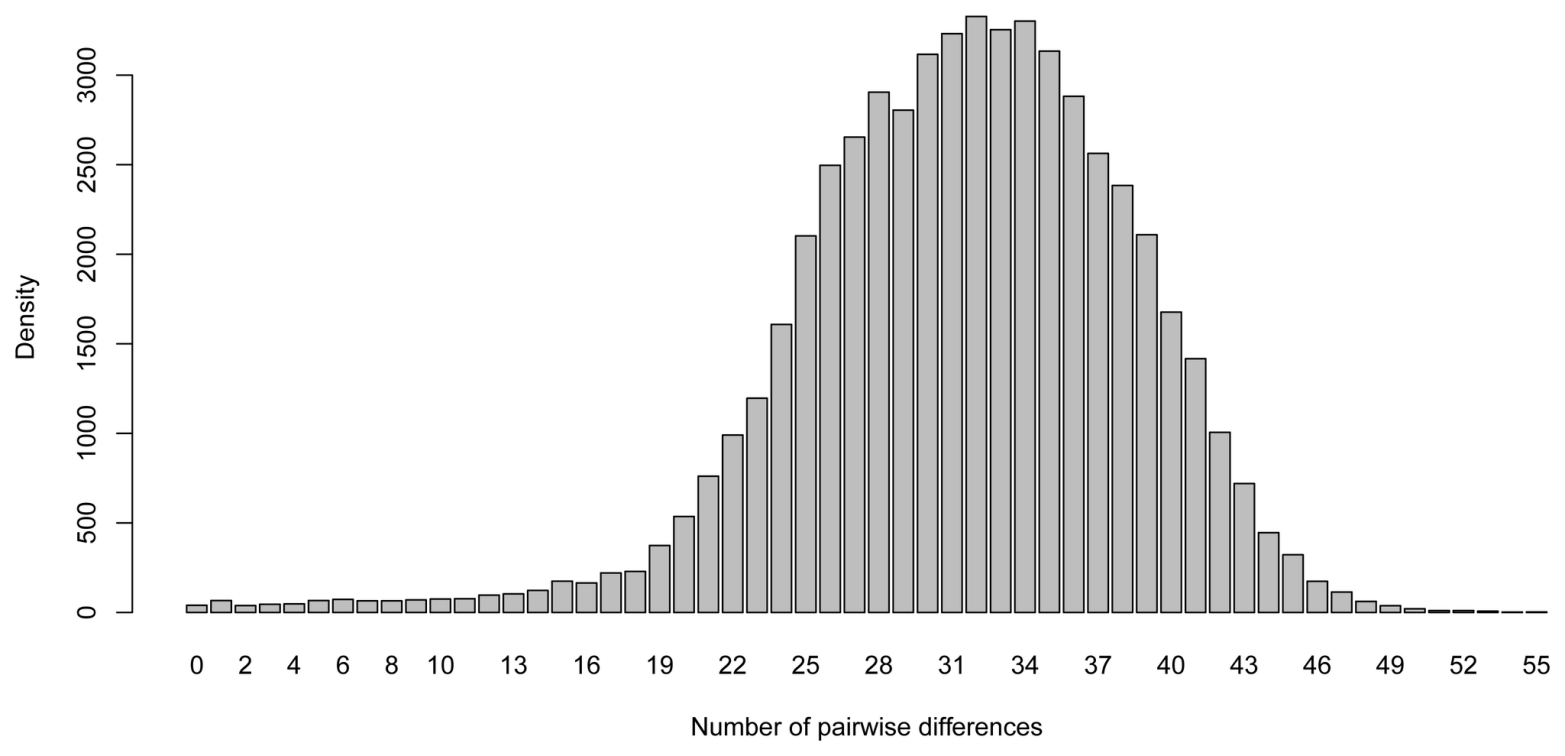
Distribution of the number of pairwise differences (in base pairs) for the target sequence among 334 mammal species.

The mammal reference DNA sequences were further compared for all species in pairs, to count the number of nucleotides that differed between the sequences of different species. Figure 2 displays the distribution of the number of nucleotide differences for the target amplicon with an overall mean number of pairwise differences of 31.5 (SD = 7). For the purpose of this method it was, however, of most interest to measure the ability to separate different species. Based on the reference sequences from the 334 different mammal species, only 0.06 % of the pairwise comparisons resulted in identical reference sequences. Thus, over 99.9 % of the species could be distinguished using the present assay. Species with identical sequences are listed in Table S4. Furthermore, a tree based on the total number of pairwise differences, for the complete target sequence, is shown in Figure S1. Closely related species tend to cluster, with only a few nucleotide differences for the complete target.

### Verification of the universal primers

The ability of the universal PCR primer pair to amplify DNA was tested with samples from a variety of different species using PCR amplification followed by Sanger sequencing. DNA sequences were successfully obtained for all 15 mammal species tested (Table 1). These sequences were confirmed to match the corresponding reference sequences in GenBank.

### Species determination of mixed samples

Table 2 presents the result from experiments with artificial mixed DNA-samples of either 1:1 or 1:99 mtDNA copy number ratios. All species were successfully detected in the 1:1 mixtures, while for some of the 1:99 mixtures, only the DNA from the species in majority was observed (Table 2). For the non-human DNA mixtures, the ratio of the obtained reads was similar to the expected ratio, although large variations were seen (Table S2). The mixtures that included DNA from human resulted, however, in a decreased number of human reads, approximately with a factor of 10-20 times in comparison with the expected ratio (Table S2). 

### Error rates

Since the reference sequences were known, experiments were performed in order to estimate error rates based on the DNA sequences obtained from the analyses. In the first test with DNA from elk, *Alces alces*, a total of 11,683 reads were obtained. 10,742 of these reads (89.6 %) were identical with the reference sequence for elk, whereas the remaining 1,211 reads (10.4 %) consisted of 211 sequence variants. The mean length of these sequences was 72 bases, thus the error rate was estimated to be 0.0014 errors per base. 

The error rate is known to be sequence specific, especially for sequences containing homo polymers when using 454 sequencing chemistry [18]. The human, *Homo sapiens*, target reference sequence contains a stretch of five adenosine bases. The second experiment, with DNA from human, resulted in 5,268 reads of which only 66 % were identical with the reference human sequence and out of the erroneous sequences 78 % consisted of a sequence variant with four adenosine bases. In total, the error rate was estimated to 0.0047 per base, thus almost five times higher than for the elk experiment. As discussed in more detail below single nucleotide errors are, however, not important for the purpose of the method described here since the complete sequence of the target amplicon is used for the search and matching.

### Samples from authentic forensic casework

**Figure 3:**
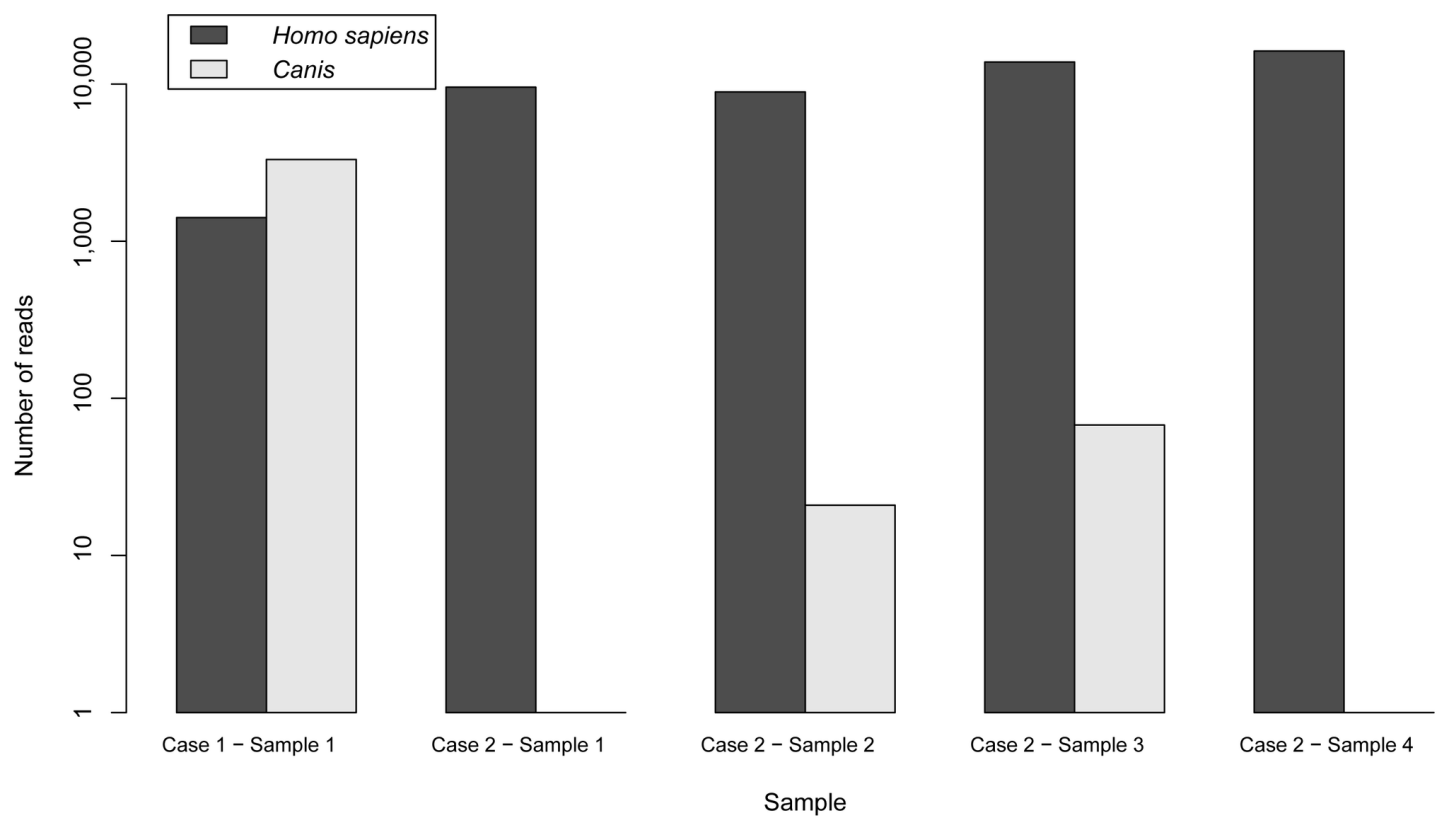
Results from experiments with DNA samples from authentic forensic cases using the 454 deep sequencing method. The number of reads obtained after analyses of five samples from authentic forensic casework are presented. Case 1 was a sample taken from a human accused of animal cruelty and case 2 was four samples taken from a human corpse with multiple bite marks.

In the first authentic case, which consisted of a man accused of animal cruelty, DNA from both human, *Homo sapiens*, and the genus *Canis* (including the subspecies domestic dog and wolf) was detected in the sample (Figure 3 In the second case, the four different samplings from bite marks, were analyzed in one single 454 sequencing run with equal input of PCR amplicons. DNA from human, *Homo sapiens*, was detected in all samples and DNA identical to the reference sequence for the genus *Canis* was additionally found in two of the samples (Figure 3). The presence of DNA from *Canis* was consistent with other findings in both cases. However, a notable observation was that the presented deep sequencing method enabled detection of small proportions of the minor component (<1 %). 

##  Discussion

Our main goal with this work was to develop a universal method for species determination including the ability to detect all species in samples of mixed DNA from different mammal species. When it comes to species determination, a large number of different DNA typing methods and case issues have been presented, see 23 for a review. The NGS method, with the universal target approach, that we here present has several advantages compared to other typing methods. For example a metagenomic approach might be too inefficient, for the purposes of the method described here, if the analyzed sample contains a major proportion of sequences from bacteria, fungi, algae or unknown species origin [1]. Methods that are species-specific could be useful when having information of what to look for, especially in mixed samples where the species of interest is present in very small amounts. In most contemporary applications one-species-specific methodologies do not, however, allow for simultaneous detection of different DNA components in a mixture. In these methods there must be an a priori hypothesis of what to look for. If casework requires the ability to detect a large variety of different species, specific assays for each species would be needed, making the task tedious, costly and not always applicable compared with having a universal method. There are, however, species-specific methods for the identification of species within a mixture. Tobe et al. [24] designed and validated an assay to identify 18 European mammal species from mixtures. Although limited to a number of specified species, the assay offers both a fast and inexpensive alternative to the herein presented assay, especially when prior information of the unknown species might be available. A universal approach, on the other hand, has the major advantage that no a priori knowledge is needed for a case. Such a method does, however, require more work in designing the target sequence and additional tests to verify that the method really is capable of detecting all the species it is aimed for.

Both the phylogenetic study of reference sequences and the verification tests of DNA of known origin showed that the method presented in this paper is suitable for the vast majority of mammal species. Even though the choice of target (16S rRNA) is less variable than other commonly used genes (e.g. cytochrome oxidase 1 and cytochrome b [25]), the target has earlier proved to be useful for species identification [16,26]. For our assay, the target satisfied the need for homologous primer sites enclosing a short variable region of only approximately 100 base pairs. The short target sequence is well suited for analysis of degraded DNA which has been shown in previous work on artificially degraded samples [19]. This is often the case, since the material in question might come from processed animal tissue, as in food, or from forensic stains destroyed by environmental conditions, or archaeological findings. Too short sequences could, however, give less phylogenetic resolution [14] and further tests might be needed in order to verify the method’s validity for species not included in this study. By redesigning the PCR primers, for example by utilising degenerated primers, the method described herein could be expanded to work for additional species to be detected. Phylogenetic studies (data not shown) of reference DNA sequences from almost 1,600 species belonging to the taxa *Vertebrate* showed for example that approximately 80 % of the species had differences of two nucleotides or less in the primer sequence regions.

The target sequence was, for most instances, proven to be variable enough to resolve the vast majority of the 334 reference mammal species used in the phylogenetic analysis 1 should, however, note that the analysis performed did not take any intraspecific variation into account. Tobe et al. [27] previously showed, in a comprehensive study, the relationship between the interspecific and the intraspecific variation for two other commonly used target genes in mtDNA. From our phylogenetic studies (Figure S1) the degree of interspecific variation revealed that the chosen target might be sensitive for intraspecific variation for some of the closely related species (for example species belonging to the genus *Cervus*). Thus, further phylogenetic studies of the intraspecific variation are advised to maximize the confidence of a given hit at the species or subspecies level. 

The main advantage using NGS deep sequencing is the ability to individually sequence each PCR amplicon at a certain depth. This increases the ability to detect traces of DNA within a mixture of species present only in small amounts. In our experimental setup of artificial DNA mixtures with different ratios, both species were always detected for the 1:1 copy number ratios. The minor component of only 1 % could be detected in all cases where the minor component was of non-human origin. Our tests indicated that the human DNA sequence was less efficiently (tenfold) detected in a mixture. The reason for this was not clear but the sequencing part of the analysis can be excluded since we obtain maximum (or very close to) number of reads from those analyses. Also the initial PCR amplification can be excluded since analysis of mixtures using standard pyrosequencing [19] result in pyrograms with expected peak heights. This leaves us with the emPCR step, which hypothetically, for unknown reasons, does not amplify the human sequences as efficiently as the other sequences when present in a mixture. Although this is a technical drawback it can actually be regarded as an advantage in casework where the human component might disturb the detection of a minor component of another species but not vice versa. 

The analysis of the samples from the two forensic cases indicated that the method also works well for authentic case samples and not only for artificially “clean” samples. In both cases DNA from *Canis* could be detected, which was as expected given other information in the cases. 

In general our experiments show that using quality requirements of no less than 20 reads for the minor DNA component is robust enough to detect the minor component down to as little as 1 % of the total DNA. Thus the method is sensitive enough to also be vulnerable to contamination. Strict routines for separating the pre- and emPCR amplification are needed and the use of dedicated barcode tags for each case can aid in monitoring possible run to run contamination. Further studies are, however, needed to monitor background levels for casework samples and to set up analysis parameters for the bioinformatic evaluation of the massive sequence data. For the latter, one must address issues of separating a true sequence variant (e.g. heteroplasmy) from a false sequence variant (e.g. amplification error, sequencing error). For the application discussed here, the complete sequence of the target is used for the identification of species, thus single base errors are not vital unless a presumable mixture contains DNA from closely related species.

In summary, we present a universal method for species identification of mammals using a targeted massively parallel sequencing approach. Both phylogenetic studies and sequencing experiments confirm the specificity of the universal primer set. Minor DNA components down to 1 % were shown to be detectable in species mixtures using the deep 454 sequencing method. Although promising results were obtained with the current settings, the rapid development of bench top instruments will further improve the method with less “hands-on”, lower detection limit and fewer sequencing errors. 

## Supporting Information

Figure S1 **Phylogenetic tree based on the targeted sequence data for 334 mammal species.** The tree was created based on pairwise difference distances among the mammal species using a UPGMA tree-making method. The branch length represents the total number of pairwise differences for the complete target region.
(PDF)

Table S1 **List of species included in the phylogenic analysis.**
(PDF)

Table S2 **Summary of the mixture analyses.**
(PDF)

Table S3 **Barcodes used in the primers of the 454 deep sequencing method.**
(PDF)

Table S4 **List of species with identical sequences for the target region.**
(PDF)
