# Supplementary figures and images for: A Universal Method for Species Identification of Mammals Utilizing Next Generation Sequencing for the Analysis of DNA Mixtures
Source: PLoS One. 2013 Dec 16;8(12):e83761. doi: 10.1371/journal.pone.0083761 (PMC3865308; doi:10.1371/journal.pone.0083761)

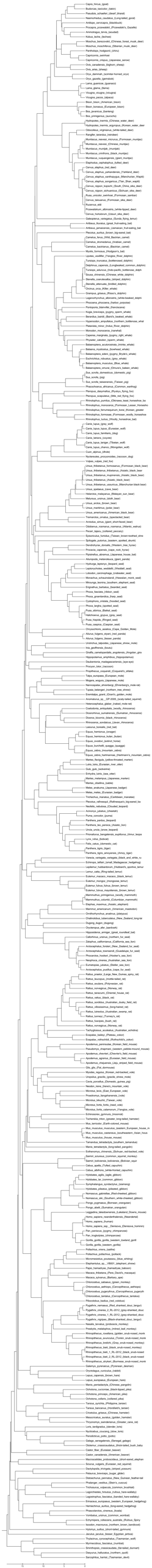

Supplement: Figure S1 — Phylogenetic tree based on the targeted sequence data for 334 mammal species. The tree was created based on pairwise difference distances among the mammal species using a UPGMA tree-making method. The branch length represents the total number of pairwise differences for the complete target region. (PDF) [file pone.0083761.s001.pdf]
