# Supplementary material for: A Universal Method for Species Identification of Mammals Utilizing Next Generation Sequencing for the Analysis of DNA Mixtures
Source: PLoS One. 2013 Dec 16;8(12):e83761. doi: 10.1371/journal.pone.0083761 (PMC3865308; doi:10.1371/journal.pone.0083761)
Supplement: Table S1 — List of species included in the phylogenic analysis. (PDF) [file pone.0083761.s002.pdf]

Table S1. Reference mammal species

Species	GenBank ID
Castor fiber (Eurasian beaver)	GenBank_acc="NC_015072
Pollachius virens (saithe)	GenBank_acc="NC_015094
Pollachius pollachius (pollack)	GenBank_acc="NC_015097
Castor canadensis (American beaver)	GenBank_acc="NC_015108
Vulpes vulpes (red fox)	GenBank_acc="NC_008434
Zalophus californianus (California sea lion)	GenBank_acc="NC_008416
Leptonychotes weddellii (Weddell seal)	GenBank_acc="NC_008424
Mirounga leonina (southern elephant seal)	GenBank_acc="NC_008422
Pusa hispida (Ringed seal)	GenBank_acc="NC_008433
Pusa sibirica (Baikal seal)	GenBank_acc="NC_008432
Hydrurga leptonyx (leopard seal)	GenBank_acc="NC_008425
Monachus schauinslandi (Hawaiian monk seal)	GenBank_acc="NC_008421
Callorhinus ursinus (northern fur seal)	GenBank_acc="NC_008415
Phocarctos hookeri (Hooker's sea lion)	GenBank_acc="NC_008418
Arctocephalus pusillus (cape fur seal)	GenBank_acc="NC_008417
Phoca fasciata (ribbon seal)	GenBank_acc="NC_008428
Phoca groenlandica (harp seal)	GenBank_acc="NC_008429
Phoca largha (spotted seal)	GenBank_acc="NC_008430
Cystophora cristata (hooded seal)	GenBank_acc="NC_008427
Erignathus barbatus (bearded seal)	GenBank_acc="NC_008426
Lobodon carcinophaga (crabeater seal)	GenBank_acc="NC_008423
Arctocephalus townsendi (Guadalupe fur seal)	GenBank_acc="NC_008420
Neophoca cinerea (Australian sea lion)	GenBank_acc="NC_008419
Pusa caspica (Caspian seal)	GenBank_acc="NC_008431
Gulo gulo (wolverine)	GenBank_acc="NC_009685
Canis lupus lupus (Eurasian wolf)	GenBank_acc="NC_009686
Trichechus manatus (Caribbean manatee)	GenBank_acc="NC_010302
Daubentonia madagascariensis (aye-aye)	GenBank_acc="NC_010299
Dendrohyrax dorsalis (Western tree hyrax)	GenBank_acc="NC_010301
Eremitalpa granti (Grant's golden mole)	GenBank_acc="NC_010304
Ursus spelaeus (cave bear)	GenBank_acc="NC_011112
Arctodus simus (giant short-faced bear)	GenBank_acc="NC_011116
Ailurus fulgens (lesser panda)	GenBank_acc="NC_011124
Meles meles (Eurasian badger)	GenBank_acc="NC_011125
Homo sapiens neanderthalensis (Neandertal)	GenBank_acc="NC_011137
Homo sapiens ssp. Denisova (Denisova hominin)	GenBank_acc="NC_013993
Micromesistius poutassou (blue whiting)	GenBank_acc="NC_015102
Rattus norvegicus (Norway rat)	GenBank_acc="NC_001665
Homo sapiens (human)	GenBank_acc="NC_012920AC_000021
Mus musculus (house mouse)	GenBank_acc="NC_005089
Mus terricolor (ESpch-colored mouse)	GenBank_acc="NC_010650
Lagostrophus fasciatus (banded hare-wallaby)	GenBank_acc="NC_008447
Eulemur mongoz (mongoose lemur)	GenBank_acc="NC_010300
Hylomys suillus (short-tailed gymnure)	GenBank_acc="NC_010298
Spilogale putorius (eastern spotted skunk)	GenBank_acc="NC_010497
Sus scrofa (pig)	GenBank_acc="NC_000845
Cavia porcellus (Domestic guinea pig)	GenBank_acc="NC_000884
Hippopotamus amphibius (hippopotamus)	GenBank_acc="NC_000889
Ornithorhynchus anatinus (platypus)	GenBank_acc="NC_000891
Balaenoptera musculus (Blue whale)	GenBank_acc="NC_001601
Halichoerus grypus (gray seal)	GenBank_acc="NC_001602

Pan troglodytes (chimpanzee)	GenBank_acc="NC_001643
Gorilla gorilla (western gorilla)	GenBank_acc="NC_001645
Glis glis (Fat dormouse)	GenBank_acc="NC_001892
Oryctolagus cuniculus (rabbit)	GenBank_acc="NC_001913
Ovis aries (sheep)	GenBank_acc="NC_001941
Spcibeus jamaicensis (Jamaican fruit-eating bat)	GenBank_acc="NC_002009
Orycteropus afer (aardvark)	GenBank_acc="NC_002078
Hylobates lar (common gibbon)	GenBank_acc="NC_002082
Sciurus vulgaris (Eurasian red squirrel)	GenBank_acc="NC_002369
Talpa europaea (European mole)	GenBank_acc="NC_002391
Physeter catodon (sperm whale)	GenBank_acc="NC_002503
Vicugna pacos (alpaca)	GenBank_acc="NC_002504
Tupaia belangeri (northern tree shrew)	GenBank_acc="NC_002521
Pteropus dasymallus (Ryukyu flying fox)	GenBank_acc="NC_002612
Pteropus scapulatus (little red flying fox)	GenBank_acc="NC_002619
Chalinolobus tuberculatus (New Zealand long-tailed	GenBank_acc="NC_002626
Echinops telfairi (small Madagascar hedgehog)	GenBank_acc="NC_002631
Thryonomys swinderianus (Greater cane rat)	GenBank_acc="NC_002658
Isoodon macrourus (northern brown bandicoot)	GenBank_acc="NC_002746
Cebus albifrons (white-fronted capuchin)	GenBank_acc="NC_002763
Macaca sylvanus (Barbary ape)	GenBank_acc="NC_002764
Nycticebus coucang (slow loris)	GenBank_acc="NC_002765
Echinosorex gymnura (moonrat)	GenBank_acc="NC_002808
Tarsius bancanus (Horsfield's tarsier)	GenBank_acc="NC_002811
Ochotona collaris (collared pika)	GenBank_acc="NC_003033
Trichosurus vulpecula (common brushtail)	GenBank_acc="NC_003039
Episoriculus fumidus (Taiwan brown-toothed shrew)	GenBank_acc="NC_003040
Microtus kikuchii (Taiwan vole)	GenBank_acc="NC_003041
Dugong dugon (dugong)	GenBank_acc="NC_003314
Vombatus ursinus (common wombat)	GenBank_acc="NC_003322
Ursus americanus (American black bear)	GenBank_acc="NC_003426
Ursus arctos (brown bear)	GenBank_acc="NC_003427
Ursus maritimus (polar bear)	GenBank_acc="NC_003428
Lemur catta (Ring-tailed lemur)	GenBank_acc="NC_004025
Macroscelides proboscideus (short-eared elephant	GenBank_acc="NC_004026
Manis tetradactyla (long-tailed pangolin)	GenBank_acc="NC_004027
Lepus europaeus (European hare)	GenBank_acc="NC_004028
Odobenus rosmarus rosmarus (Atlantic walrus)	GenBank_acc="NC_004029
Eumetopias jubatus (Steller sea lion)	GenBank_acc="NC_004030
Galeopterus variegatus (Sunda flying lemur)	GenBank_acc="NC_004031
Tamandua tetradactyla (southern tamandua)	GenBank_acc="NC_004032
Muntiacus reevesi (Chinese muntjac)	GenBank_acc="NC_004069
Muntiacus muntjak (muntjak)	GenBank_acc="NC_004563
Procavia capensis (cape rock hyrax)	GenBank_acc="NC_004919
Chrysochloris asiatica (Cape Golden Mole)	GenBank_acc="NC_004920
Elephantulus sp. VB001 (elephant shrew)	GenBank_acc="NC_004921
Hemiechinus auritus (long-eared hedgehog)	GenBank_acc="NC_005033
Mogera wogura (Japanese mole)	GenBank_acc="NC_005035
Acinonyx jubatus (cheetah)	GenBank_acc="NC_005212
Balaena mysticetus (bowhead whale)	GenBank_acc="NC_005268
Caperea marginata (pygmy right whale)	GenBank_acc="NC_005269
Eschrichtius robustus (grey whale)	GenBank_acc="NC_005270
Balaenoptera acutorostrata (minke whale)	GenBank_acc="NC_005271

Kogia breviceps (pygmy sperm whale)	GenBank_acc="NC_005272
Hyperoodon ampullatus (northern bottlenose whale)	GenBank_acc="NC_005273
Berardius bairdii (Baird's beaked whale)	GenBank_acc="NC_005274
Platanista minor (Indus River dolphin)	GenBank_acc="NC_005275
Inia geoffrensis (boutu)	GenBank_acc="NC_005276
Pontoporia blainvillei (franciscana)	GenBank_acc="NC_005277
Lagenorhynchus albirostris (white-beaked dolphin)	GenBank_acc="NC_005278
Monodon monoceros (narwhal)	GenBank_acc="NC_005279
Jaculus jaculus (lesser Egyptian jerboa)	GenBank_acc="NC_005314
Ochotona princeps (American pika)	GenBank_acc="NC_005358
Rhinolophus pumilus (Okinawa least horseshoe bat)	GenBank_acc="NC_005434
Ursus thibetanus ussuricus (Manchurian black bear)	GenBank_acc="NC_011117
Apodemus agrarius (Eurasian field mouse)	GenBank_acc="NC_016428
Pongo pygmaeus (Bornean orangutan)	GenBank_acc="NC_001646
Canis lupus laniger (Tibetan wolf)	GenBank_acc="NC_011218
Puma concolor (puma)	GenBank_acc="NC_016470
Rattus norvegicus (Norway rat)	GenBank_acc="AC_000022
Pan paniscus (pygmy chimpanzee)	GenBank_acc="NC_001644
Felis catus (domestic cat)	GenBank_acc="NC_001700
Papio hamadryas (hamadryas baboon)	GenBank_acc="NC_001992
Canis lupus familiaris (dog)	GenBank_acc="NC_002008
Erinaceus europaeus (western European hedgehog)	GenBank_acc="NC_002080
Pongo abelii (Sumatran orangutan)	GenBank_acc="NC_002083
Tachyglossus aculeatus (Australian echidna)	GenBank_acc="NC_003321
Arctocephalus forsteri (New Zealand fur seal)	GenBank_acc="NC_004023
Muntiacus crinifrons (black muntjac)	GenBank_acc="NC_004577
Urotrichus talpoides (Japanese shrew mole)	GenBank_acc="NC_005034
Capra hircus (goat)	GenBank_acc="NC_005044
Elephas maximus (Asiatic elephant)	GenBank_acc="NC_005129
Phocoena phocoena (harbor porpoise)	GenBank_acc="NC_005280
Nannospalax ehrenbergi (Ehrenberg's mole-rat)	GenBank_acc="NC_005315
Rhinolophus monoceros (Formosan Lesser Horseshoe Bat)	GenBank_acc="NC_005433
Pipistrellus abramus (Japanese house bat)	GenBank_acc="NC_005436
Chlorocebus aethiops (Cercopithecus aethiops)	GenBank_acc="NC_007009
Pantholops hodgsonii (chiru)	GenBank_acc="NC_007441
Mammuthus primigenius (woolly mammoth)	GenBank_acc="NC_007596
Lipotes vexillifer (Yangtze River dolphin)	GenBank_acc="NC_007629
Dasyurus hallucatus (northern quoll)	GenBank_acc="NC_007630
Sminthopsis crassicaudata (fat-tailed dunnSpc)	GenBank_acc="NC_007631
Echymipera rufescens australis (Rufous Spiny	GenBank_acc="NC_007632
Cervus elaphus (red deer)	GenBank_acc="NC_007704
Cricetulus griseus (Chinese hamster)	GenBank_acc="NC_007936
Balaenoptera omurai (Omura's baleen whale)	GenBank_acc="NC_007937
Balaenoptera edeni (pygmy Bryde's whale)	GenBank_acc="NC_007938
Microtus levis (East European vole)	GenBank_acc="NC_008064
Chlorocebus sabaeus (green monkey)	GenBank_acc="NC_008066
Canis lupus (gray wolf)	GenBank_acc="NC_008092
Canis latrans (coyote)	GenBank_acc="NC_008093
Phascolarctos cinereus (koala)	GenBank_acc="NC_008133
Dactylopsila trivirgata (striped possum)	GenBank_acc="NC_008134
Petaurus breviceps (sugar glider)	GenBank_acc="NC_008135
Lagorchestes hirsutus (rufous hare-wallaby)	GenBank_acc="NC_008136
Phalanger vestitus (Stein's cuscus)	GenBank_acc="NC_008137

Distoechurus pennatus (New Guinean feather-tailed	GenBank_acc="NC_008145
Galemys pyrenaicus (Pyrenean desman)	GenBank_acc="NC_008156
Nasalis larvatus (proboscis monkey)	GenBank_acc="NC_008216
Presbytis melalophos (mitred leaf monkey)	GenBank_acc="NC_008217
Rhinopithecus roxellana (golden snub-nosed monkey)	GenBank_acc="NC_008218
Piliocolobus badius (red colobus)	GenBank_acc="NC_008219
Pygathrix nemaeus (Red shanked douc langur)	GenBank_acc="NC_008220
Rusa unicolor swinhoei (Formosan sambar)	GenBank_acc="NC_008414
Neofelis nebulosa (Clouded leopard)	GenBank_acc="NC_008450
Cervus taiouanus (Formosan sika deer)	GenBank_acc="NC_008462
Muntiacus reevesi micrurus (Formosan muntjac)	GenBank_acc="NC_008491
Elaphodus cephalophus (tufted deer)	GenBank_acc="NC_008749
Ursus thibetanus mupinensis (Asiatic black bear)	GenBank_acc="NC_008753
Phacochoerus africanus (Common wSpchog)	GenBank_acc="NC_008830
Anomalurus sp. GP-2005 (scaly-tailed squirrel)	GenBank_acc="NC_009056
Procyon lotor (raccoon)	GenBank_acc="NC_009126
Ursus thibetanus formosanus (Formosan black bear)	GenBank_acc="NC_009331
Ailuropoda melanoleuca (giant panda)	GenBank_acc="NC_009492
Ammotragus lervia (aoudad)	GenBank_acc="NC_009510
Mammut americanum (American mastodon)	GenBank_acc="NC_009574
Camelus bactrianus (Bactrian camel)	GenBank_acc="NC_009628
Camelus ferus (Wild Bactrian camel)	GenBank_acc="NC_009629
Meles anakuma (Japanese badger)	GenBank_acc="NC_009677
MSpces melampus (Japanese mSpcen)	GenBank_acc="NC_009678
Ailurus fulgens styani (red panda)	GenBank_acc="NC_009691
Enhydra lutris (sea otter)	GenBank_acc="NC_009692
Chlorocebus pygerythrus (Cercopithecus pygerythrus)	GenBank_acc="NC_009747
Chlorocebus tantalus (Cercopithecus tantalus)	GenBank_acc="NC_009748
Camelus dromedarius (Arabian camel)	GenBank_acc="NC_009849
Helarctos malayanus (Malayan sun bear)	GenBank_acc="NC_009968
Tremarctos ornatus (spectacled bear)	GenBank_acc="NC_009969
Melursus ursinus (sloth bear)	GenBank_acc="NC_009970
Ursus thibetanus (Asiatic black bear)	GenBank_acc="NC_009971
Mus musculus musculus (eastern European house mouse)	GenBank_acc="NC_010339
Canis lupus chanco (Mongolian wolf)	GenBank_acc="NC_010340
Uncia uncia (snow leopard)	GenBank_acc="NC_010638
Capricornis swinhoei	GenBank_acc="NC_010640
Panthera pardus (leopard)	GenBank_acc="NC_010641
Panthera tigris (tiger)	GenBank_acc="NC_010642
Ochotona curzoniae (black-lipped pika)	GenBank_acc="NC_011029
Propithecus coquereli (Coquerel's sifaka)	GenBank_acc="NC_011053
Ursus thibetanus thibetanus (Asiatic black bear)	GenBank_acc="NC_011118
Gorilla gorilla gorilla (western lowland gorilla)	GenBank_acc="NC_011120
Rhinolophus formosae (Formosan woolly horseshoe bat)	GenBank_acc="NC_011304
Lutra lutra (Eurasian river otter)	GenBank_acc="NC_011358
Macaca thibetana (Pere David's macaque)	GenBank_acc="NC_011519
MSpces zibellina (sable)	GenBank_acc="NC_011579
Rattus tanezumi (Oriental house rat)	GenBank_acc="NC_011638
Hydropotes inermis (Chinese water deer)	GenBank_acc="NC_011821
Lama guanicoe (guanaco)	GenBank_acc="NC_011822
Thylacinus cynocephalus (Tasmanian wolf)	GenBank_acc="NC_011944
Myrmecobius fasciatus (numbat)	GenBank_acc="NC_011949
Stenella attenuata (bridled dolphin)	GenBank_acc="NC_012051

Stenella coeruleoalba (striped dolphin)	GenBank_acc="NC_012053
Sousa chinensis (Chinese white dolphin)	GenBank_acc="NC_012057
Tursiops aduncus (Indo-pacific bottlenose dolphin)	GenBank_acc="NC_012058
Tursiops truncatus (bottlenosed dolphin)	GenBank_acc="NC_012059
Delphinus capensis (Longbeaked common dolphin)	GenBank_acc="NC_012061
Grampus griseus (Risso's dolphin)	GenBank_acc="NC_012062
Sus scrofa domesticus (domestic pig)	GenBank_acc="NC_012095
Capricornis crispus (Japanese serow)	GenBank_acc="NC_012096
Antilope cervicapra (blackbuck)	GenBank_acc="NC_012098
Giraffa camelopardalis angolensis (Angolan giraffe)	GenBank_acc="NC_012100
Lama glama (llama)	GenBank_acc="NC_012102
Pecari tajacu (collared peccary)	GenBank_acc="NC_012103
MSpces flavigula (yellow-throated mSpcen)	GenBank_acc="NC_012141
Bison bison (American bison)	GenBank_acc="NC_012346
Mus musculus castaneus (southeastern Asian house	GenBank_acc="NC_012387
Coelodonta antiquitatis (woolly rhinoceros)	GenBank_acc="NC_012681
Diceros bicornis (black rhinoceros)	GenBank_acc="NC_012682
Rhinoceros sondaicus (Javan rhinoceros)	GenBank_acc="NC_012683
Dicerorhinus sumatrensis (Sumatran rhinoceros)	GenBank_acc="NC_012684
Moschus berezovskii (Chinese forest musk deer)	GenBank_acc="NC_012694
Bos javanicus (banteng)	GenBank_acc="NC_012706
Galago senegalensis (Senegal galago)	GenBank_acc="NC_012761
Otolemur crassicaudatus (thick-tailed bush baby)	GenBank_acc="NC_012762
Loris tardigradus (slender loris)	GenBank_acc="NC_012763
Perodicticus potto (potto)	GenBank_acc="NC_012764
Eulemur fulvus fulvus (brown lemur)	GenBank_acc="NC_012766
Eulemur fulvus mayottensis (brown lemur)	GenBank_acc="NC_012769
Eulemur macaco macaco (black lemur)	GenBank_acc="NC_012771
Varecia variegata variegata (black and white ruffled	GenBank_acc="NC_012773
Tarsius syrichta (Philippine tarsier)	GenBank_acc="NC_012774
Saimiri sciureus (common squirrel monkey)	GenBank_acc="NC_012775
Tscherskia triton (greater long-tailed hamster)	GenBank_acc="NC_013068
Budorcas taxicolor (takin)	GenBank_acc="NC_013069
Mesocricetus auratus (golden hamster)	GenBank_acc="NC_013276
Cuon alpinus (dhole)	GenBank_acc="NC_013445
Vicugna vicugna (vicugna)	GenBank_acc="NC_013558
Proedromys liangshanensis (vole)	GenBank_acc="NC_013563
Eothenomys chinensis (Sichuan red-backed vole)	GenBank_acc="NC_013571
Nyctereutes procyonoides (raccoon dog)	GenBank_acc="NC_013700
Naemorhedus caudatus (Long-tailed goral)	GenBank_acc="NC_013751
Moschus moschiferus (Siberian musk deer)	GenBank_acc="NC_013753
Cervus hortulorum (Ussuri sika deer)	GenBank_acc="NC_013834
Cervus elaphus xanthopygus (Manchurian Wapiti)	GenBank_acc="NC_013836
Cervus elaphus yarkandensis (Yarkland deer)	GenBank_acc="NC_013840
Bos primigenius (aurochs)	GenBank_acc="NC_013996
Hylobates agilis (agile gibbon)	GenBank_acc="NC_014042
Bison bonasus (European bison)	GenBank_acc="NC_014044
Hylobates pileatus (pileated gibbon)	GenBank_acc="NC_014045
Symphalangus syndactylus (siamang)	GenBank_acc="NC_014047
Nomascus siki (Southern white-cheeked gibbon)	GenBank_acc="NC_014051
Lepilemur hubbardorum (Hubbard's sportive lemur)	GenBank_acc="NC_014453
Lynx rufus (bobcat)	GenBank_acc="NC_014456
Orcinus orca (Killer whale)	GenBank_acc="NC_014682

Sus scrofa taiwanensis (Taiwan pig)	GenBank_acc="NC_014692
Leggadina lakedownensis (Lakeland Downs mouse)	GenBank_acc="NC_014696
Pseudomys chapmani (western pebble-mound mouse)	GenBank_acc="NC_014698
Rucervus eldi	GenBank_acc="NC_014701
Cervus elaphus songaricus (Tian Shan wapiti)	GenBank_acc="NC_014703
Panthera tigris amoyensis (Amoy tiger)	GenBank_acc="NC_014770
Procapra przewalskii (Przewalski's Gazelle)	GenBank_acc="NC_014875
Heterocephalus glaber (naked mole-rat)	GenBank_acc="NC_015112
Microtus fortis fortis (reed vole)	GenBank_acc="NC_015241
Microtus fortis calamorum (Yangtze vole)	GenBank_acc="NC_015243
Odocoileus virginianus (white-tailed deer)	GenBank_acc="NC_015247
Plecotus auritus (brown big-eared bat)	GenBank_acc="NC_015484
Rhinopithecus avunculus (Tonkin snub-nosed monkey)	GenBank_acc="NC_015485
Rhinopithecus bieti (black snub-nosed monkey)	GenBank_acc="NC_015486
Mammuthus columbi (Columbian mammoth)	GenBank_acc="NC_015529
Lepus capensis (brown hare)	GenBank_acc="NC_015841
Ovis canadensis (bighorn sheep)	GenBank_acc="NC_015889
Manis pentadactyla (Chinese pangolin)	GenBank_acc="NC_016008
Neodon irene (Irene's mountain vole)	GenBank_acc="NC_016055
Apodemus peninsulae (Korean field mouse)	GenBank_acc="NC_016060
Equus hemionus (onager)	GenBank_acc="NC_016061
Cervus nippon kopschi (South China sika deer)	GenBank_acc="NC_016178
Prionailurus bengalensis euptilurus (Amur leopard	GenBank_acc="NC_016189
Rhinolophus ferrumequinum korai (Korean greater	GenBank_acc="NC_016191
Oryx dammah (scimitar-horned oryx)	GenBank_acc="NC_016421
Oryx gazella (gemsbok)	GenBank_acc="NC_016422
Myodes regulus (Korean red-backed vole)	GenBank_acc="NC_016427
Apodemus chejuensis (Jeju striped field mouse)	GenBank_acc="NC_016662
Cebus apella (Tufted capuchin)	GenBank_acc="NC_016666
Pseudois schaeferi (dwarf bharal)	GenBank_acc="NC_016689
Przewalskium albirostris (white-lipped deer)	GenBank_acc="NC_016707
Spcibeus lituratus (great fruit-eating bat)	GenBank_acc="NC_016871
Plecotus rafinesquii (Rafinesque's big-eared bat)	GenBank_acc="NC_016872
Lasiurus borealis (red bat)	GenBank_acc="NC_016873
Muntiacus vuquangensis (giant muntjac)	GenBank_acc="NC_016920
Apodemus chevrieri (Chevrier's field mouse)	GenBank_acc="NC_017599
Hydropotes inermis argyropus (Korean water deer)	GenBank_acc="NC_018032
Panthera leo persica (Asiatic lion)	GenBank_acc="NC_018053
Rhinopithecus brelichi (Gray snub-nosed monkey)	GenBank_acc="NC_018057
Rhinopithecus bieti 1 RL-2012 (black snub-nosed	GenBank_acc="NC_018058
Rhinopithecus strykeri (Burmese snub-nosed monkey)	GenBank_acc="NC_018059
Rhinopithecus bieti 2 RL-2012 (black snub-nosed	GenBank_acc="NC_018060
Pygathrix nigripes (Black-shanked douc langur)	GenBank_acc="NC_018061
Pygathrix cinerea 1 RL-2012 (gray-shanked douc	GenBank_acc="NC_018062
Pygathrix cinerea 2 RL-2012 (gray-shanked douc	GenBank_acc="NC_018063
Saimiri boliviensis boliviensis (Bolivian squirrel	GenBank_acc="NC_018096
Eospalax baileyi (Plateau zokor)	GenBank_acc="NC_018098
Eospalax rothschildi (Rothschild's zokor)	GenBank_acc="NC_018535
Rhinolophus luctus (Woolly horseshoe bat)	GenBank_acc="NC_018539
Hipposideros armiger (great roundleaf bat)	GenBank_acc="NC_018540
Cervus nippon sichuanicus (Sichuan sika deer)	GenBank_acc="NC_018595
Uropsilus gracilis (gracile shrew mole)	GenBank_acc="NC_018598
Kobus leche (lechwe)	GenBank_acc="NC_018603

Nomascus gabriellae (Red-cheeked gibbon)	GenBank_acc="NC_018753
Equus zebra (mountain zebra)	GenBank_acc="NC_018779
Equus zebra hSpcmannae (HSpcmann's mountain zebra)	GenBank_acc="NC_018780
Equus burchellii quagga (quagga)	GenBank_acc="NC_018781
Equus hemionus kulan (kulan)	GenBank_acc="NC_018782
Equus ovodovi (extinct horse)	GenBank_acc="NC_018783
Sarcophilus harrisii (Tasmanian devil)	GenBank_acc="NC_018788
Rattus rattus (black rat)	GenBank_acc="NC_012374
Rattus exulans (Polynesian rat)	GenBank_acc="NC_012389
Rattus praetor (Large New Guinea spiny rat)	GenBank_acc="NC_012461
Rattus leucopus (mottle-tailed rat)	GenBank_acc="NC_014855
Rattus lutreolus (Australian swamp rat)	GenBank_acc="NC_014858
Rattus tunneyi (Tunney's rat)	GenBank_acc="NC_014861
Rattus villosissimus (long-haired rat)	GenBank_acc="NC_014864
Rattus fuscipes (bush rat)	GenBank_acc="NC_014867
Rattus sordidus (Australian dusky field rat)	GenBank_acc="NC_014871
Rangifer tarandus (reindeer)	GenBank_acc="NC_007703
Myotis formosus (Hodgson's bat)	GenBank_acc="NC_015828
