# Supplementary material for: A Universal Method for Species Identification of Mammals Utilizing Next Generation Sequencing for the Analysis of DNA Mixtures
Source: PLoS One. 2013 Dec 16;8(12):e83761. doi: 10.1371/journal.pone.0083761 (PMC3865308; doi:10.1371/journal.pone.0083761)
Supplement: Table S2 — Summary of the mixture analyses. (PDF) [file pone.0083761.s003.pdf]

Table S2. Summary of mixture analyis using 454 deep sequencing

Species 1:Species 2	Expected mixed ratio (sp1:sp2)	Observed mixed ratio (sp1:sp2)	Number of reads after filtering	Comments
Dog:Human	1:1	16:1	1,645	
Dog:Human	99:1	480:1	2,405	Minor component found with less than 20 reads
Elk:Human	1:1	68:1	2,958	
Elk:Human	99:1	Elk	3,069	
Deer:Elk	1:1	1:2	2,105	
Deer:Elk	99:1	415:1	1,665	Minor component found with less than 20 reads
Bear:Human	1:1	7:1	772	
Bear:Human	99:1	Bear	1,474	
Wild boar:Human	1:1	8:1	1,559	
Wild boar:Human	99:1	Wildboar	1,392	
Elk:Human	99:1	1245:1	18,689	Minor component found with less than 20 reads
Human:Wild boar	99:1	7:1	15,711	
Elk:Human	1:1	19:1	23,055	
Elk:Human	99:1	Elk	11,165	
Human:Wild boar	99:1	5:1	7,420	
Elk:Human	1:1	23:1	11,527	
Roe deer: Elk:Bear:Dog	1:1:1:1	74:18:9:1	6,594	
Elk: Dog	99:1	308:1	16,354	
Dog:Bear	99:1	26:1	22,473	
Bear: Elk	99:1	52:1	32,975	
Cow:Pig	99:1	1591:1	92,352	

Dog (*Canis lupus familiaris*), Human (*Homo sapiens*), Elk (*Alces Alces*), Deer (*Dama dama*), Bear (*Ursus arctos*), Wild boar (*Sus scrofa*), Roe deer (*Capreolus capreolus*), Pig (*Sus scrofa domesticus*), Cow (*Bos taurus*)
