# Supplementary material for: A Universal Method for Species Identification of Mammals Utilizing Next Generation Sequencing for the Analysis of DNA Mixtures
Source: PLoS One. 2013 Dec 16;8(12):e83761. doi: 10.1371/journal.pone.0083761 (PMC3865308; doi:10.1371/journal.pone.0083761)
Supplement: Table S3 — Barcodes used in the primers of the 454 deep sequencing method. (PDF) [file pone.0083761.s004.pdf]

Table S3. Barcodes used for 454 sequencing

acgagtgcgt
acgctcgaca
agacgcactc
agcactgtag
atatcgcgag
atcagacacg
cgtgtctcta
ctcgcgtgtc
tagtatcagc
tctctatgcg
