# Supplementary material for: A Universal Method for Species Identification of Mammals Utilizing Next Generation Sequencing for the Analysis of DNA Mixtures
Source: PLoS One. 2013 Dec 16;8(12):e83761. doi: 10.1371/journal.pone.0083761 (PMC3865308; doi:10.1371/journal.pone.0083761)
Supplement: Table S4 — List of species with identical sequences for the target region. (PDF) [file pone.0083761.s005.pdf]

Table S4. Species with identical DNA sequences for the target region

Species 1	Species 2
Canis lupus laniger (Tibetan wolf)	Canis lupus chanco (Mongolian wolf)
Pusa sibirica (Baikal seal)	Halichoerus grypus (gray seal)
Martes melampus (Japanese marten)	Martes zibellina (sable)
Ursus thibetanus formosanus (Formosan black bear)	Ursus thibetanus thibetanus (Asiatic black bear)
Ursus thibetanus formosanus (Formosan black bear)	Ursus thibetanus mupinensis (Asiatic black bear)
Ursus thibetanus thibetanus (Asiatic black bear)	Ursus thibetanus mupinensis (Asiatic black bear)
Panthera tigris (tiger)	Panthera tigris amoyensis (Amoy tiger)
Equus zebra (mountain zebra)	Equus zebra hartmannae (Hartmann's mountain zebra)
Sus scrofa (pig)	Sus scrofa domesticus (domestic pig)
Stenella attenuata (bridled dolphin)	Orcinus orca (Killer whale)
Tursiops aduncus (Indo-pacific bottlenose dolphin)	Delphinus capensis (Longbeaked common dolphin)
Tursiops aduncus (Indo-pacific bottlenose dolphin)	Tursiops truncatus (bottlenosed dolphin)
Tursiops aduncus (Indo-pacific bottlenose dolphin)	Sousa chinensis (Chinese white dolphin)
Delphinus capensis (Longbeaked common dolphin)	Tursiops truncatus (bottlenosed dolphin)
Delphinus capensis (Longbeaked common dolphin)	Sousa chinensis (Chinese white dolphin)
Tursiops truncatus (bottlenosed dolphin)	Sousa chinensis (Chinese white dolphin)
Muntiacus reevesi (Chinese muntjac)	Muntiacus reevesi micrurus (Formosan muntjac)
Cervus elaphus xanthopygus (Manchurian Wapiti)	Cervus elaphus songaricus (Tian Shan wapiti)
Cervus nippon kopschi (South China sika deer)	Cervus nippon sichuanicus (Sichuan sika deer)
Rusa unicolor swinhoei (Formosan sambar)	Cervus taiouanus (Formosan sika deer)
Rusa unicolor swinhoei (Formosan sambar)	Przewalskium albirostris (white-lipped deer)
Rusa unicolor swinhoei (Formosan sambar)	Rucervus eldi
Cervus taiouanus (Formosan sika deer)	Przewalskium albirostris (white-lipped deer)
Cervus taiouanus (Formosan sika deer)	Rucervus eldi
Przewalskium albirostris (white-lipped deer)	Rucervus eldi
Hydropotes inermis (Chinese water deer)	Hydropotes inermis argyropus (Korean water deer)
Camelus bactrianus (Bactrian camel)	Camelus ferus (Wild Bactrian camel)
Camelus bactrianus (Bactrian camel)	Camelus dromedarius (Arabian camel)
Camelus ferus (Wild Bactrian camel)	Camelus dromedarius (Arabian camel)
Mammuthus primigenius (woolly mammoth)	Mammuthus columbi (Columbian mammoth)
Eulemur fulvus fulvus (brown lemur)	Eulemur fulvus mayottensis (brown lemur)
Homo sapiens neanderthalensis (Neandertal)	Homo sapiens (human)
Chlorocebus pygerythrus (Cercopithecus pygerythrus)	Chlorocebus tantalus (Cercopithecus tantalus)
Rhinopithecus bieti (black snub-nosed monkey)	Rhinopithecus bieti 1 RL-2012 (black snub-nosed
Saimiri sciureus (common squirrel monkey)	Saimiri boliviensis boliviensis (Bolivian squirrel
